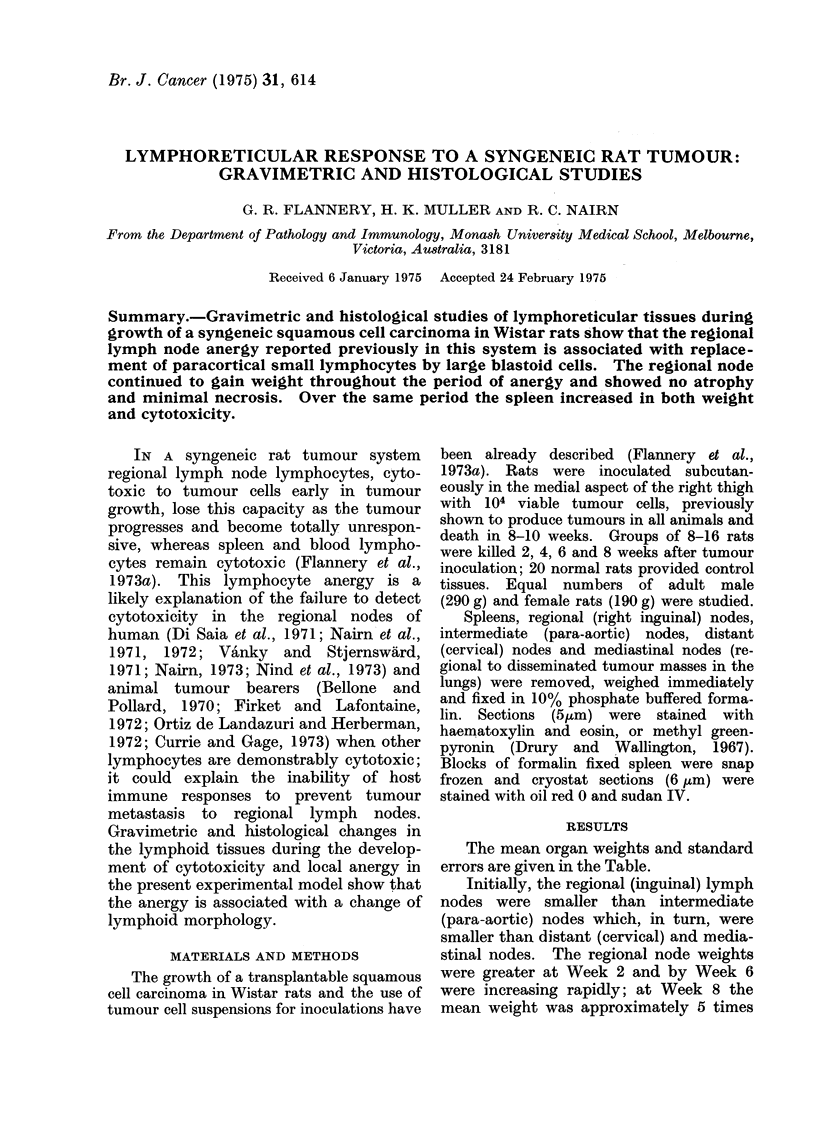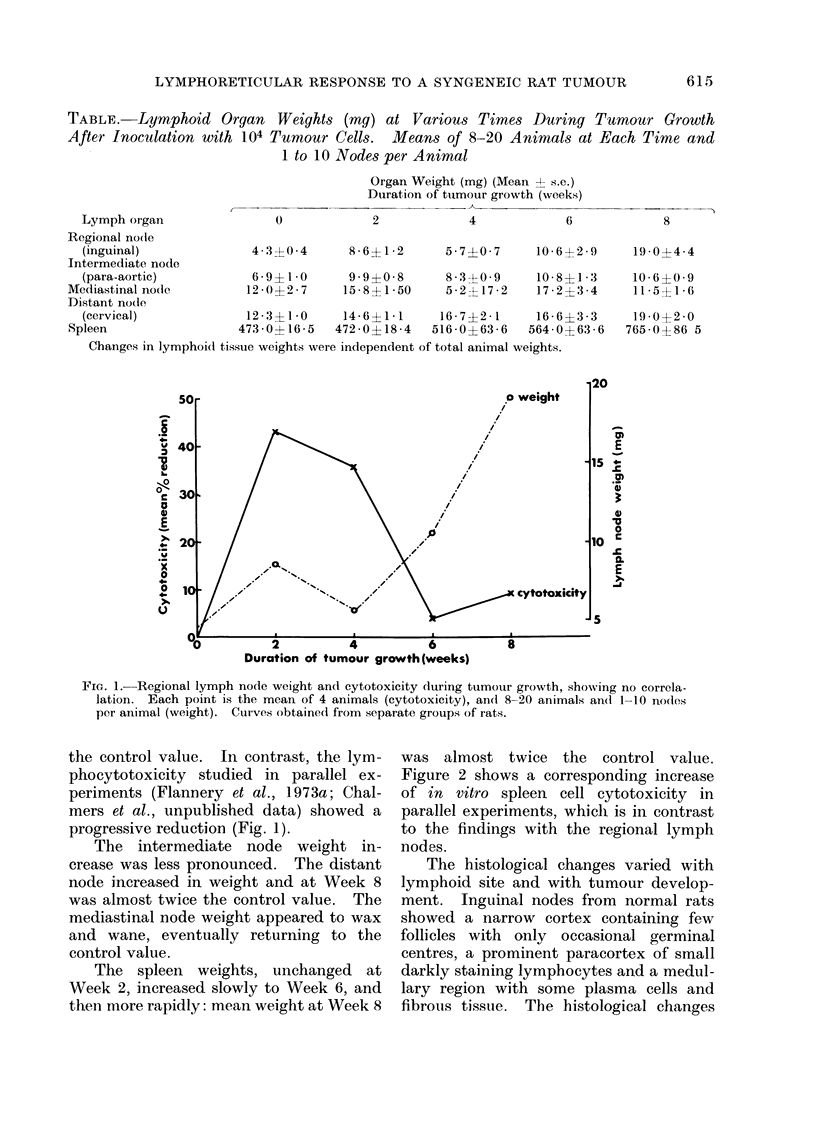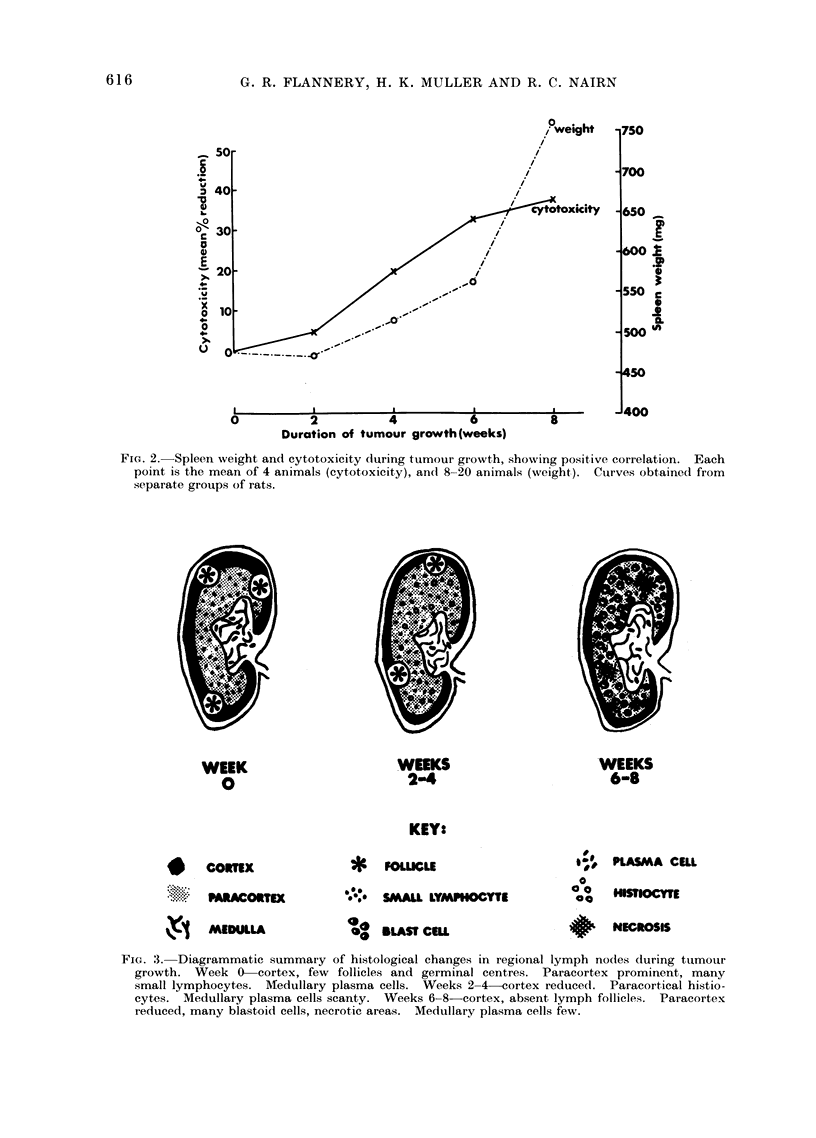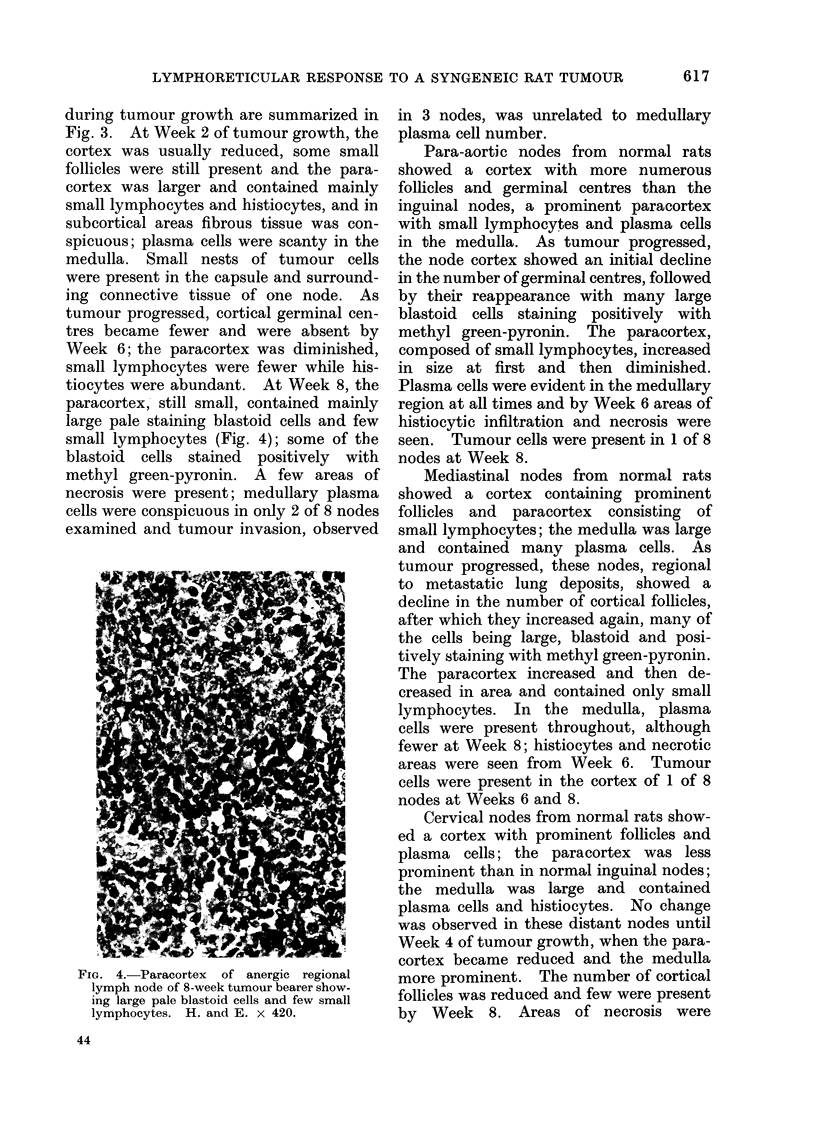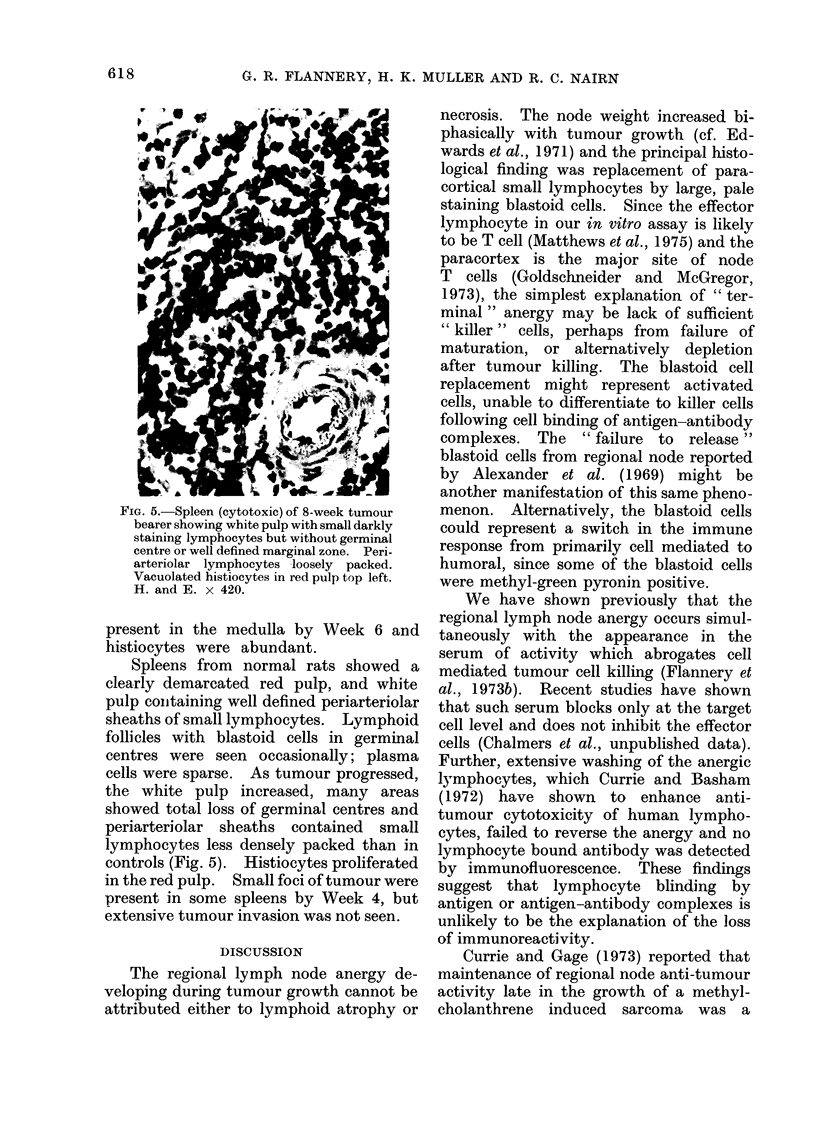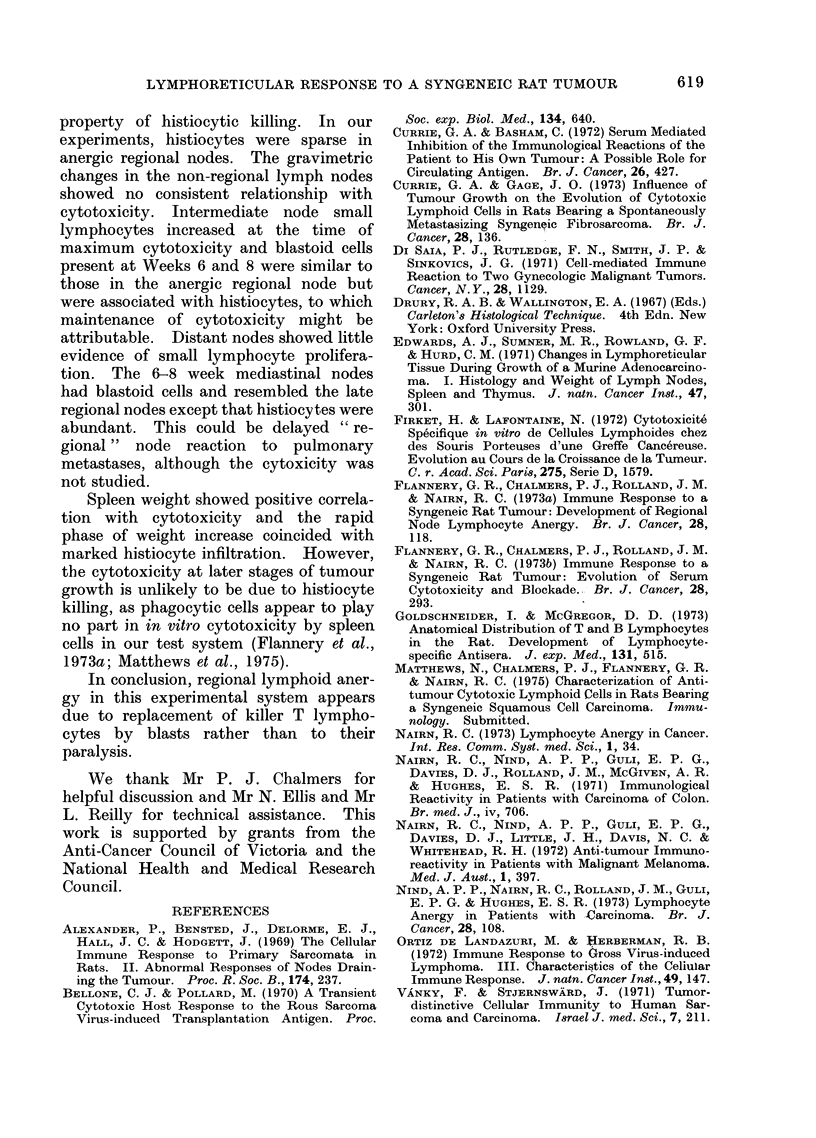# Lymphoreticular response to a syngeneic rat tumour: gravimetric and histological studies.

**DOI:** 10.1038/bjc.1975.107

**Published:** 1975-06

**Authors:** G. R. Flannery, H. K. Muller, R. C. Nairn

## Abstract

**Images:**


*Br. J. Cancer* (1975) **31**, 614

### LYMPHORETICULAR RESPONSE TO A SYNGENEIC RAT TUMOUR: GRAVIMETRIC AND HISTOLOGICAL STUDIES

G. R. FLANNERY, H. K. MULLER AND R. C. NAIRN

*From the Department of Pathology and Immunology, Monash University Medical School, Melbourne, Victoria, Australia,* 3181

Received 6 January 1975 Accepted 24 February 1975

**Summary.—Gravimetric and histological studies of lymphoreticular tissues during growth of a syngeneic squamous cell carcinoma in Wistar rats show that the regional lymph node anergy reported previously in this system is associated with replacement of paracortical small lymphocytes by large blastoid cells. The regional node continued to gain weight throughout the period of anergy and showed no atrophy and minimal necrosis. Over the same period the spleen increased in both weight and cytotoxicity.**

IN A syngeneic rat tumour system regional lymph node lymphocytes, cytotoxic to tumour cells early in tumour growth, lose this capacity as the tumour progresses and become totally unresponsive, whereas spleen and blood lymphocytes remain cytotoxic (Flannery *et al.*, 1973*a*). This lymphocyte anergy is a likely explanation of the failure to detect cytotoxicity in the regional nodes of human (Di Saia *et al.*, 1971; Nairn *et al.*, 1971, 1972; Vánky and Stjernswärd, 1971; Nairn, 1973; Nind *et al.*, 1973) and animal tumour bearers (Bellone and Pollard, 1970; Firket and Lafontaine, 1972; Ortiz de Landazuri and Herberman, 1972; Currie and Gage, 1973) when other lymphocytes are demonstrably cytotoxic; it could explain the inability of host immune responses to prevent tumour metastasis to regional lymph nodes. Gravimetric and histological changes in the lymphoid tissues during the development of cytotoxicity and local anergy in the present experimental model show that the anergy is associated with a change of lymphoid morphology.

### MATERIALS AND METHODS

The growth of a transplantable squamous cell carcinoma in Wistar rats and the use of tumour cell suspensions for inoculations have been already described (Flannery *et al.*, 1973*a*). Rats were inoculated subcutaneously in the medial aspect of the right thigh with $10^4$ viable tumour cells, previously shown to produce tumours in all animals and death in 8–10 weeks. Groups of 8–16 rats were killed 2, 4, 6 and 8 weeks after tumour inoculation; 20 normal rats provided control tissues. Equal numbers of adult male (290 g) and female rats (190 g) were studied.

Spleens, regional (right inguinal) nodes, intermediate (para-aortic) nodes, distant (cervical) nodes and mediastinal nodes (regional to disseminated tumour masses in the lungs) were removed, weighed immediately and fixed in 10% phosphate buffered formalin. Sections (5$\mu$m) were stained with haematoxylin and eosin, or methyl green-pyronin (Drury and Wallington, 1967). Blocks of formalin fixed spleen were snap frozen and cryostat sections (6 $\mu$m) were stained with oil red 0 and sudan IV.

### RESULTS

The mean organ weights and standard errors are given in the Table.

Initially, the regional (inguinal) lymph nodes were smaller than intermediate (para-aortic) nodes which, in turn, were smaller than distant (cervical) and mediastinal nodes. The regional node weights were greater at Week 2 and by Week 6 were increasing rapidly; at Week 8 the mean weight was approximately 5 times

TABLE.—*Lymphoid Organ Weights (mg) at Various Times During Tumour Growth After Inoculation with* $10^4$ *Tumour Cells. Means of* 8–20 *Animals at Each Time and* 1 *to* 10 *Nodes per Animal*

| Lymph organ | Organ Weight (mg) (Mean ± s.e.) Duration of tumour growth (weeks) 0 | 2 | 4 | 6 | 8 |
|---|---|---|---|---|---|
| Regional node (inguinal) | 4·3±0·4 | 8·6±1·2 | 5·7±0·7 | 10·6±2·9 | 19·0±4·4 |
| Intermediate node (para-aortic) | 6·9±1·0 | 9·9±0·8 | 8·3±0·9 | 10·8±1·3 | 10·6±0·9 |
| Mediastinal node | 12·0±2·7 | 15·8±1·50 | 5·2±17·2 | 17·2±3·4 | 11·5±1·6 |
| Distant node (cervical) | 12·3±1·0 | 14·6±1·1 | 16·7±2·1 | 16·6±3·3 | 19·0±2·0 |
| Spleen | 473·0±16·5 | 472·0±18·4 | 516·0±63·6 | 564·0±63·6 | 765·0±86 5 |

Changes in lymphoid tissue weights were independent of total animal weights.

FIG. 1.—Regional lymph node weight and cytotoxicity during tumour growth, showing no correlation. Each point is the mean of 4 animals (cytotoxicity), and 8–20 animals and 1–10 nodes per animal (weight). Curves obtained from separate groups of rats.

the control value. In contrast, the lymphocytotoxicity studied in parallel experiments (Flannery *et al.*, 1973*a*; Chalmers *et al.*, unpublished data) showed a progressive reduction (Fig. 1).

The intermediate node weight increase was less pronounced. The distant node increased in weight and at Week 8 was almost twice the control value. The mediastinal node weight appeared to wax and wane, eventually returning to the control value.

The spleen weights, unchanged at Week 2, increased slowly to Week 6, and then more rapidly: mean weight at Week 8 was almost twice the control value. Figure 2 shows a corresponding increase of *in vitro* spleen cell cytotoxicity in parallel experiments, which is in contrast to the findings with the regional lymph nodes.

The histological changes varied with lymphoid site and with tumour development. Inguinal nodes from normal rats showed a narrow cortex containing few follicles with only occasional germinal centres, a prominent paracortex of small darkly staining lymphocytes and a medullary region with some plasma cells and fibrous tissue. The histological changes

FIG. 2.—Spleen weight and cytotoxicity during tumour growth, showing positive correlation. Each point is the mean of 4 animals (cytotoxicity), and 8–20 animals (weight). Curves obtained from separate groups of rats.

FIG. 3.—Diagrammatic summary of histological changes in regional lymph nodes during tumour growth. Week 0—cortex, few follicles and germinal centres. Paracortex prominent, many small lymphocytes. Medullary plasma cells. Weeks 2–4—cortex reduced. Paracortical histiocytes. Medullary plasma cells scanty. Weeks 6–8—cortex, absent lymph follicles. Paracortex reduced, many blastoid cells, necrotic areas. Medullary plasma cells few.

during tumour growth are summarized in Fig. 3. At Week 2 of tumour growth, the cortex was usually reduced, some small follicles were still present and the paracortex was larger and contained mainly small lymphocytes and histiocytes, and in subcortical areas fibrous tissue was conspicuous; plasma cells were scanty in the medulla. Small nests of tumour cells were present in the capsule and surrounding connective tissue of one node. As tumour progressed, cortical germinal centres became fewer and were absent by Week 6; the paracortex was diminished, small lymphocytes were fewer while histiocytes were abundant. At Week 8, the paracortex, still small, contained mainly large pale staining blastoid cells and few small lymphocytes (Fig. 4); some of the blastoid cells stained positively with methyl green-pyronin. A few areas of necrosis were present; medullary plasma cells were conspicuous in only 2 of 8 nodes examined and tumour invasion, observed in 3 nodes, was unrelated to medullary plasma cell number.

FIG. 4.—Paracortex of anergic regional lymph node of 8-week tumour bearer showing large pale blastoid cells and few small lymphocytes. H. and E. × 420.

Para-aortic nodes from normal rats showed a cortex with more numerous follicles and germinal centres than the inguinal nodes, a prominent paracortex with small lymphocytes and plasma cells in the medulla. As tumour progressed, the node cortex showed an initial decline in the number of germinal centres, followed by their reappearance with many large blastoid cells staining positively with methyl green-pyronin. The paracortex, composed of small lymphocytes, increased in size at first and then diminished. Plasma cells were evident in the medullary region at all times and by Week 6 areas of histiocytic infiltration and necrosis were seen. Tumour cells were present in 1 of 8 nodes at Week 8.

Mediastinal nodes from normal rats showed a cortex containing prominent follicles and paracortex consisting of small lymphocytes; the medulla was large and contained many plasma cells. As tumour progressed, these nodes, regional to metastatic lung deposits, showed a decline in the number of cortical follicles, after which they increased again, many of the cells being large, blastoid and positively staining with methyl green-pyronin. The paracortex increased and then decreased in area and contained only small lymphocytes. In the medulla, plasma cells were present throughout, although fewer at Week 8; histiocytes and necrotic areas were seen from Week 6. Tumour cells were present in the cortex of 1 of 8 nodes at Weeks 6 and 8.

Cervical nodes from normal rats showed a cortex with prominent follicles and plasma cells; the paracortex was less prominent than in normal inguinal nodes; the medulla was large and contained plasma cells and histiocytes. No change was observed in these distant nodes until Week 4 of tumour growth, when the paracortex became reduced and the medulla more prominent. The number of cortical follicles was reduced and few were present by Week 8. Areas of necrosis were

FIG. 5.—Spleen (cytotoxic) of 8-week tumour bearer showing white pulp with small darkly staining lymphocytes but without germinal centre or well defined marginal zone. Periarteriolar lymphocytes loosely packed. Vacuolated histiocytes in red pulp top left. H. and E. × 420.

present in the medulla by Week 6 and histiocytes were abundant.

Spleens from normal rats showed a clearly demarcated red pulp, and white pulp containing well defined periarteriolar sheaths of small lymphocytes. Lymphoid follicles with blastoid cells in germinal centres were seen occasionally; plasma cells were sparse. As tumour progressed, the white pulp increased, many areas showed total loss of germinal centres and periarteriolar sheaths contained small lymphocytes less densely packed than in controls (Fig. 5). Histiocytes proliferated in the red pulp. Small foci of tumour were present in some spleens by Week 4, but extensive tumour invasion was not seen.

### DISCUSSION

The regional lymph node anergy developing during tumour growth cannot be attributed either to lymphoid atrophy or necrosis. The node weight increased biphasically with tumour growth (cf. Edwards *et al.*, 1971) and the principal histological finding was replacement of paracortical small lymphocytes by large, pale staining blastoid cells. Since the effector lymphocyte in our *in vitro* assay is likely to be T cell (Matthews *et al.*, 1975) and the paracortex is the major site of node T cells (Goldschneider and McGregor, 1973), the simplest explanation of " terminal " anergy may be lack of sufficient " killer " cells, perhaps from failure of maturation, or alternatively depletion after tumour killing. The blastoid cell replacement might represent activated cells, unable to differentiate to killer cells following cell binding of antigen–antibody complexes. The " failure to release " blastoid cells from regional node reported by Alexander *et al.* (1969) might be another manifestation of this same phenomenon. Alternatively, the blastoid cells could represent a switch in the immune response from primarily cell mediated to humoral, since some of the blastoid cells were methyl-green pyronin positive.

We have shown previously that the regional lymph node anergy occurs simultaneously with the appearance in the serum of activity which abrogates cell mediated tumour cell killing (Flannery *et al.*, 1973*b*). Recent studies have shown that such serum blocks only at the target cell level and does not inhibit the effector cells (Chalmers *et al.*, unpublished data). Further, extensive washing of the anergic lymphocytes, which Currie and Basham (1972) have shown to enhance antitumour cytotoxicity of human lymphocytes, failed to reverse the anergy and no lymphocyte bound antibody was detected by immunofluorescence. These findings suggest that lymphocyte blinding by antigen or antigen–antibody complexes is unlikely to be the explanation of the loss of immunoreactivity.

Currie and Gage (1973) reported that maintenance of regional node anti-tumour activity late in the growth of a methylcholanthrene induced sarcoma was a property of histiocytic killing. In our experiments, histiocytes were sparse in anergic regional nodes. The gravimetric changes in the non-regional lymph nodes showed no consistent relationship with cytotoxicity. Intermediate node small lymphocytes increased at the time of maximum cytotoxicity and blastoid cells present at Weeks 6 and 8 were similar to those in the anergic regional node but were associated with histiocytes, to which maintenance of cytotoxicity might be attributable. Distant nodes showed little evidence of small lymphocyte proliferation. The 6–8 week mediastinal nodes had blastoid cells and resembled the late regional nodes except that histiocytes were abundant. This could be delayed "regional" node reaction to pulmonary metastases, although the cytoxicity was not studied.

Spleen weight showed positive correlation with cytotoxicity and the rapid phase of weight increase coincided with marked histiocyte infiltration. However, the cytotoxicity at later stages of tumour growth is unlikely to be due to histiocyte killing, as phagocytic cells appear to play no part in *in vitro* cytotoxicity by spleen cells in our test system (Flannery *et al.*, 1973*a*; Matthews *et al.*, 1975).

In conclusion, regional lymphoid anergy in this experimental system appears due to replacement of killer T lymphocytes by blasts rather than to their paralysis.

We thank Mr P. J. Chalmers for helpful discussion and Mr N. Ellis and Mr L. Reilly for technical assistance. This work is supported by grants from the Anti-Cancer Council of Victoria and the National Health and Medical Research Council.

### REFERENCES

Alexander, P., Bensted, J., Delorme, E. J., Hall, J. C. & Hodgett, J. (1969) The Cellular Immune Response to Primary Sarcomata in Rats. II. Abnormal Responses of Nodes Draining the Tumour. *Proc. R. Soc. B.*, **174**, 237.

Bellone, C. J. & Pollard, M. (1970) A Transient Cytotoxic Host Response to the Rous Sarcoma Virus-induced Transplantation Antigen. *Proc. Soc. exp. Biol. Med.*, **134**, 640.

Currie, G. A. & Basham, C. (1972) Serum Mediated Inhibition of the Immunological Reactions of the Patient to His Own Tumour: A Possible Role for Circulating Antigen. *Br. J. Cancer*, **26**, 427.

Currie, G. A. & Gage, J. O. (1973) Influence of Tumour Growth on the Evolution of Cytotoxic Lymphoid Cells in Rats Bearing a Spontaneously Metastasizing Syngeneic Fibrosarcoma. *Br. J. Cancer*, **28**, 136.

Di Saia, P. J., Rutledge, F. N., Smith, J. P. & Sinkovics, J. G. (1971) Cell-mediated Immune Reaction to Two Gynecologic Malignant Tumors. *Cancer, N.Y.*, **28**, 1129.

Drury, R. A. B. & Wallington, E. A. (1967) (Eds.) *Carleton's Histological Technique*. 4th Edn. New York: Oxford University Press.

Edwards, A. J., Sumner, M. R., Rowland, G. F. & Hurd, C. M. (1971) Changes in Lymphoreticular Tissue During Growth of a Murine Adenocarcinoma. I. Histology and Weight of Lymph Nodes, Spleen and Thymus. *J. natn. Cancer Inst.*, **47**, 301.

Firket, H. & Lafontaine, N. (1972) Cytotoxicité Spécifique *in vitro* de Cellules Lymphoides chez des Souris Porteuses d'une Greffe Cancéreuse. Evolution au Cours de la Croissance de la Tumeur. *C. r. Acad. Sci. Paris*, **275**, Serie D, 1579.

Flannery, G. R., Chalmers, P. J., Rolland, J. M. & Nairn, R. C. (1973*a*) Immune Response to a Syngeneic Rat Tumour: Development of Regional Node Lymphocyte Anergy. *Br. J. Cancer*, **28**, 118.

Flannery, G. R., Chalmers, P. J., Rolland, J. M. & Nairn, R. C. (1973*b*) Immune Response to a Syngeneic Rat Tumour: Evolution of Serum Cytotoxicity and Blockade. *Br. J. Cancer*, **28**, 293.

Goldschneider, I. & McGregor, D. D. (1973) Anatomical Distribution of T and B Lymphocytes in the Rat. Development of Lymphocyte-specific Antisera. *J. exp. Med.*, **131**, 515.

Matthews, N., Chalmers, P. J., Flannery, G. R. & Nairn, R. C. (1975) Characterization of Anti-tumour Cytotoxic Lymphoid Cells in Rats Bearing a Syngeneic Squamous Cell Carcinoma. *Immunology*. Submitted.

Nairn, R. C. (1973) Lymphocyte Anergy in Cancer. *Int. Res. Comm. Syst. med. Sci.*, **1**, 34.

Nairn, R. C., Nind, A. P. P., Guli, E. P. G., Davies, D. J., Rolland, J. M., McGiven, A. R. & Hughes, E. S. R. (1971) Immunological Reactivity in Patients with Carcinoma of Colon. *Br. med. J.*, iv, 706.

Nairn, R. C., Nind, A. P. P., Guli, E. P. G., Davies, D. J., Little, J. H., Davis, N. C. & Whitehead, R. H. (1972) Anti-tumour Immunoreactivity in Patients with Malignant Melanoma. *Med. J. Aust.*, **1**, 397.

Nind, A. P. P., Nairn, R. C., Rolland, J. M., Guli, E. P. G. & Hughes, E. S. R. (1973) Lymphocyte Anergy in Patients with Carcinoma. *Br. J. Cancer*, **28**, 108.

Ortiz de Landazuri, M. & Herberman, R. B. (1972) Immune Response to Gross Virus-induced Lymphoma. III. Characteristics of the Cellular Immune Response. *J. natn. Cancer Inst.*, **49**, 147.

Vánky, F. & Stjernswärd, J. (1971) Tumor-distinctive Cellular Immunity to Human Sarcoma and Carcinoma. *Israel J. med. Sci.*, **7**, 211.